# Aging Behavior of Styrene–Butadiene Rubber (SBR)-Modified Asphalt Under the Coupled Effects of Intense UV Radiation and Large Temperature Differences

**DOI:** 10.3390/ma18112527

**Published:** 2025-05-27

**Authors:** Yanling Xu, Bo Tian, Hongzhou Zhu, Junxin Wang

**Affiliations:** 1 School of Construction Engineering, Chongqing City Vocational College, Chongqing 402160, China; Fengzhongdebai_he@126.com; 2 Research Institute of Highway Ministry of Transport, Beijing 100088, China; 3 School of Civil Engineering, Chongqing Jiaotong University, Chongqing 400074, China; zhuhongzhouchina@cqjtu.edu.cn (H.Z.); meteorpretty@126.com (J.W.)

**Keywords:** SBR-modified asphalt, coupled aging, aging behavior, chemical analysis, microstructure and micromechanics analysis

## Abstract

Intense ultraviolet (UV) radiation is often accompanied by large temperature differences in high-altitude cold regions. Therefore, investigating the aging behavior of SBR asphalt under intense UV radiation and large temperature differences is crucial for prolonging the lifespan and maintenance of styrene–butadiene rubber (SBR)-modified asphalt pavements in high-altitude cold regions. This study investigated the aging process of SBR-modified asphalt by analyzing the chemical components, microstructures, and micromechanics of both base and SBR-modified asphalt under combined effects. Attenuated total reflection Fourier transform infrared spectroscopy (ATR-FTIR), gel permeation chromatography (GPC), and atomic force microscopy (AFM) were utilized to analyze this evolutionary process. The results indicated that the chemical components and microstructural properties of the SBR-modified asphalt underwent significant changes during the aging process under the combined effects of intense UV radiation and large temperature differences. The SBR-modified asphalt exhibited the same aging trend for both the chemical composition and microstructure of the matrix asphalt. However, its aging process in the SBR-modified asphalt was notably slower. This delay was primarily caused by the mesh structure of the SBR-modified asphalt, which created an initial buffer period during aging. Additionally, the degradation of SBR replenished the lost components in the asphalt colloid and inhibited the aging process. The research results indicated that the SBR-modified asphalt exhibited superior aging and cracking resistance with respect to the matrix asphalt. However, the critical cracking time for the surface cracks in the SBR-modified asphalt was earlier than that in the matrix asphalt under the combined effects. It was suggested to use the “modulus ratio” (defined as the Young’s modulus ratio of the surface asphalt layer to the underlying asphalt layer) to quantitatively assess the risk of surface cracking, with a higher modulus ratio indicating a greater risk of cracking or a higher degree of cracking.

## 1. Introduction

Aging significantly impacts the lifespan of asphalt pavements and has been widely researched in the road infrastructure industry [1]. Climate pavement adaptability is an integral part of a holistic concept of road design, construction, and pavement management. The correct design of pavement structures can protect roads from the adverse effects of underlying permafrost and enhance the overall durability of the pavement [2]. Asphalt pavements rapidly deteriorate because of aging caused by climatic and environmental factors, like temperature, oxygen, water, and ultraviolet radiation [3,4].

Laboratory methods, such as thin film oven tests (TFOTs), rolling TFOTs (RTFOTs), and pressure aging vessel (PAV) tests, have been developed to simulate the effects of both short-term and long-term aging [5]. Importantly, both aging methods involve primarily thermal oxidative aging. Conventional laboratory aging protocols overlook environmental influences like ultraviolet radiation, moisture, and temperature variations, thus failing to accurately simulate the real aging behavior of asphalt binder. Researchers have studied the impact of various environmental factors to better understand asphalt binder aging. Kemp et al. [6] conducted a study on the UV aging of asphalt concrete at a temperature of 35 °C and UV irradiance of 1000 mW/cm^2^ for 18 h. The experimental results indicated that the effect of UV on asphalt film thickness exceeded at least 5 µm. Tia et al. [7] examined the prolonged aging of asphalt mixtures, including thermo-oxidative and UV aging, utilizing a forced hot air convection aging chamber. The results indicated that UV aging was the main cause of aging, although it occurred only on the surface of asphalt concrete. Yang et al. [8] reported significant differences in the effects of thermal oxidative aging and UV aging on asphalt. As the aging time increased, the UV-aged asphalt binder became lighter in color, whereas the thermally oxidized asphalt binder became darker. Rheological analysis revealed that UV radiation significantly impacted the surface characteristics of the asphalt binder. Yang et al. [9] explored the aging processes and characteristics of asphalt when exposed to thermal oxidation, ultraviolet radiation, and moisture. The study revealed that water notably mitigated the aging acceleration effect of ultraviolet radiation, yet it expedited the growth of micro-cracks induced by ultraviolet exposure on asphalt surfaces and led to significant erosion of the asphalt binder at elevated temperatures. Guan et al. [10] studied the combined effects of ultraviolet (UV) radiation, temperature, and humidity on the rheological properties of asphalt binders. Their findings revealed that this coupled aging process increased the stiffness and high-temperature performance of the binders while simultaneously decreasing their fatigue and low-temperature performance. Xu et al. [11] studied the aging mechanism of matrix asphalt under the combined effects of intense UV and large temperature variations. They reported that asphalt exhibited asphaltene “migration” behaviors under the combined action. Hossain et al. [12] compared SBS-modified bitumen properties through different aging techniques, such as rotating film oven (RTFO), pressure aging vessel (PAV), and ultraviolet (UV) exposure. The results indicated that UV aging caused the most pronounced effects, leading to complete degradation of the polymers in SBS asphalt. Zhang et al. [13] found that UV aging caused greater degradation in SBS copolymers compared to PAV aging. Different aging factors distinctly influence the macro- and micro-properties of asphalt binders, leading to significant variations in their aging mechanisms. Owing to the coupling effects among the various environmental factors, the aging patterns and mechanisms become quite complex. Therefore, conducting in-depth research on coupled aging holds substantial theoretical importance and practical engineering value. High-altitude cold regions experience intense ultraviolet radiation along with significant diurnal temperature differences.

Asphalt materials face significant challenges in performance and durability due to harsh environmental conditions and increasing traffic volumes [14]. As a result, polymers are typically added to matrix asphalt to enhance its performance [15,16]. The challenging climate in western China necessitates the widespread use of modified asphalts, including SBS, SBR, and rubberized asphalt. SBR-modified asphalt is favored for its superior low-temperature ductility and cost-effectiveness [17,18]. Additionally, research indicates that styrene–butadiene rubber (SBR) significantly improves matrix asphalt’s resistance to ultraviolet aging [19]. Based on kinetic principles, SBR-modified asphalt has a higher activation energy and better antiaging performance than matrix asphalt [20]. The high concentration of double-bond structures in SBR makes SBR-modified asphalt vulnerable to oxidation, which disrupts the SBR–asphalt network and notably impacts the performance of the modified binder [21,22,23]. The polymer aging mechanism is more complex than that of matrix asphalt [24,25]. In high-altitude cold regions, strong UV radiation causes photo-oxidative degradation of SBR asphalt, impacting its chemical structure and physical properties. Furthermore, research indicates that large temperature differences significantly deteriorate the rheological properties and microstructure of asphalt slurries [26,27]. Understanding the aging behavior of SBR-modified asphalt under the coupled effects of strong UV and large temperature differences is crucial for the maintenance and regeneration of asphalt pavements in high-altitude cold regions. This knowledge aids in reducing maintenance costs and developing durable pavement materials.

In summary, the adverse effects of ultraviolet aging and significant temperature variations on asphalt pavement are undeniable. However, the existing research still lacks sufficient understanding of the aging mechanisms of SBR-modified asphalt under the combined influence of intense UV radiation and large temperature differences in high-altitude cold regions. Based on the climatic characteristics of the Tibet Plateau region, this study employed a custom-designed indoor coupled environmental aging system to simulate intense ultraviolet (UV) radiation, significant temperature cycling, and controlled humidity for asphalt aging experiments. This study examined the chemical composition, molecular distribution, surface microstructure, and micromechanical properties of asphalt across different aging durations to understand the aging evolution mechanism of SBR-modified asphalt under the combined effects of intense UV radiation and large temperature differences. The research results will provide a solid theoretical basis for the formulation of subsequent maintenance strategies for SBR-modified asphalt pavements and for developing innovative pavement preservation materials.

## 2. Materials and Methods

### 2.1. Experimental Material

In this work, the matrix asphalt was Shell90#, while the polymer modifier SBR was in the form of a white powder. The modified asphalt contained 4% SBR modifier. The preparation of the SBR-modified asphalt primarily referred to the Chinese standard DB21/T 2538-2015 [28]. First, the matrix was heated to 150 °C to achieve a liquid state. Subsequently, 4.0 wt% of the SBR modifier was incrementally incorporated into the matrix at a steady temperature of 160 °C and stirred at a low speed for 15 min to ensure adequate swelling. The mixture was treated with high-speed shear (4000–4500 r/min) at 165 °C for 40 min, followed by stirring at 600–700 r/min for 30 min. It was subsequently maintained at a constant temperature of 165 °C for one hour to produce the SBR-modified asphalt [29]. The basic physical indexes of the matrix asphalt, as outlined in Table 1, were conducted by reference to Chinese standards JTG-E20-2011 [30] and JTJ F40-2004 [31].

### 2.2. Coupled Aging Test

This study independently designed a combined environmental aging device, considering strong UV radiation, large temperature variations, and humidity, based on the investigation of actual environmental conditions in the Tibetan Plateau, China. The Tibetan Plateau is characterized by extremely low temperatures all year round, large temperature differences (e.g., the annual extreme maximum surface temperature difference in Tibet Province can reach 64.9 °C [32]), and the highest UV radiation intensity in the country (e.g., the average daily maximum UV radiation in Lhasa and Nagqu is greater than 72.1 W/m^2^ [33]). In this study, two 60 cm, 1000 W high-pressure mercury lamps were used to achieve a uniform sample irradiation intensity of 180 W/m^2^ (UVA + UVB). The surface temperature of the sample was maintained within a range of −20 °C to 50 °C, while the humidity for the experiment was set at 30%. The thickness of the molded asphalt binding material was set to approximately 1 mm [11]. The inner diameter of the disc was 14 cm, and approximately 16 g of asphalt was weighed according to the density of the asphalt used for molding. The indoor accelerated simulated aging times of the samples were set at 0 days (0 d), 5 days (5 d), 10 days (10 d), 15 days (15 d), and 20 days (20 d). The temperature cycling control program for the sample surface started by maintaining a constant temperature of 50 °C for 2 h, gradually decreasing the temperature to −20 °C within 1 h, holding at −20 °C for 2 h, and, finally, rapidly heating to 50 °C within 1 h. The total duration of one complete large-temperature difference cycle was 6 h. The aging times of the samples at 0 d, 5 d, 10 d, 15 d, and 20 d corresponded to 0, 20, 40, 60, and 80 large-temperature difference cycles, respectively.

### 2.3. Test Methods

#### 2.3.1. ATR-FTIR Test

This study employed ATR-FTIR to analyze the functional groups present in asphalt. Infrared spectroscopy data were obtained with a Thermo Scientific Nicolet iS50 ATR-FTIR analyzer (Waltham, MA, USA). The scanning range was set from 450 to 4000 cm^−1^, with each scan conducted 32 times at a spectral resolution of 4 cm^−1^. The sample was placed on a horizontal zinc selenide ATR crystal to facilitate multiple reflections. After each measurement, the ATR crystal was cleaned thoroughly with dichloromethane, and each sample underwent three parallel analyses to guarantee both accuracy and reliability.

#### 2.3.2. Gel Permeation Chromatography (GPC) Test

GPC was used to analyze the molecular weight and distribution of asphalt before and after coupled aging. The experiments utilized an Agilent 1260 gel permeation chromatograph with tetrahydrofuran (THF) as the mobile phase solvent (Santa Clara, CA, USA), operating at a flow rate of 1 mL/min and a temperature of 35 °C. A sample volume of 100 μL was injected each time, polystyrene was used as the standard, and each measurement was collected once.

#### 2.3.3. AFM Test

Atomic force microscopy (AFM) was employed for micro-surface imaging in tap mode and for acquiring micromechanical properties in contact mode. Surface topographic characterization was conducted using tap mode with an HQ NSC14/Pt probe (Loveland, CO, USA), which has a nominal spring constant of 5.0 N/m, a resonance frequency of about 160 Hz, and a tip radius of 8 nm. The scanning rate was set at 0.5 μm/s, with a scanning area of 20 × 20 μm^2^, and each image comprised 256 pixels. At least 5 regions were selected for scanning. In the force–distance (F-D) curve experiments, a PPP-CONT-20 probe from Nano Sensors (Neuchâtel, Switzerland) was employed in contact mode, featuring a nominal spring constant of 0.02 to 0.77 N/m, a resonance frequency of 6 to 21 Hz, and a tip radius of 8 nm. The scanning rate was 0.5 μm/s, and the actual probe stiffness needed to be calibrated thermally before each measurement to obtain accurate probe stiffness. Each sample was tested a minimum of six times, with the average value representing the maximum adhesion force. All tests were conducted at ambient conditions of 25 °C, with two replicate samples required for each test.

## 3. Results and Discussion

### 3.1. Evolution of the Sample Surface Morphology

**Figure 1:**
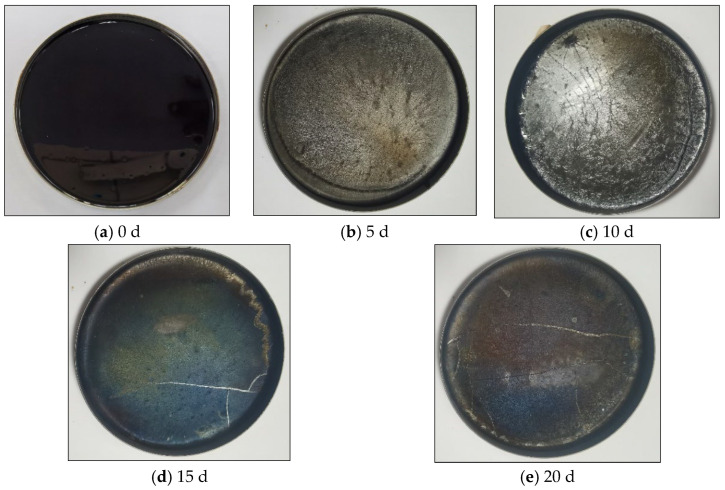
Surface morphology images of SBR asphalt at various aging intervals.

The SBR asphalt samples’ surface underwent significant alterations due to the combined effects of intense UV radiation and substantial temperature fluctuations. The changes in the surface of the SBR asphalt throughout the aging process are shown in Figure 1. Initially bright and smooth, the mirror-like surface formed a thin film with minor contraction wrinkles in the early aging stages. With aging, the contractions intensified, the surface glossiness decreased, the color deepened, and the surface dried, developing noticeable cracks in later stages. Prior studies [11] have documented the surface morphology characteristics of matrix asphalt. In comparison to the matrix asphalt, the entire surface of the SBR-modified asphalt exhibited fewer and shallower wrinkles. The surface’s localized graininess decreased over time because of the SBR modifier, as SBR was degraded during the aging process. Moreover, the cracking time of the SBR-modified asphalt occurred earlier than that of the matrix asphalt, and the specific reasons are analyzed in the subsequent content.

**Figure 2:**
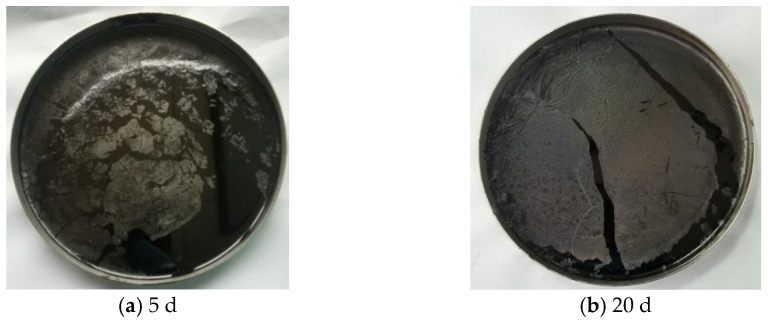
Typical representative images of the state of the aged asphalt samples after melting.

After the aged asphalt samples were heated to approximately 165 °C, the film layer did not melt, and after stirring, the samples remained insoluble in the underlying asphalt layer. Compared with the unaged asphalt, the asphalt beneath the film showed no significant visual changes. As shown in Figure 2, a notable difference in the film layer thickness was observed between the asphalt samples aged for 5 d and 20 d, indicating a continuous increase in thickness with prolonged aging. Additionally, once the asphalt was exposed to ultraviolet light, it quickly formed a film layer. The molecular structures and micromorphologies of the film layer and the underlying asphalt are analyzed in the subsequent analysis.

### 3.2. Functional Group Analysis

This section analyzes the wavenumber range from 600 cm^−1^ to 4000 cm^−1^, calculating the indices for each asphalt functional group using Equations (1)–(4) [34,35].

(1) $$I_{\mathrm{CH}=\mathrm{CH}}=\frac{A_{{966\;\mathrm{cm}}^{-1}}\;}{\sum A_{{4000\;\mathrm{cm}}^{-1}{\;\mathrm{and}\;600\;\mathrm{cm}}^{-1}}\;}$$

(2) $$I_{S=O}=\frac{A_{{1030\;\mathrm{cm}}^{-1}}\;}{\sum A_{{4000\;\mathrm{cm}}^{-1}{\;\mathrm{and}\;600\;\mathrm{cm}}^{-1}}\;}$$

(3) $$I_{C=C}=\frac{A_{{1600\;\mathrm{cm}}^{-1}}\;}{\sum A_{{4000\;\mathrm{cm}}^{-1}{\;\mathrm{and}\;600\;\mathrm{cm}}^{-1}}\;}$$

(4) $$I_{C=O}=\frac{A_{{1700\;\mathrm{cm}}^{-1}}\;}{\sum A_{{4000\;\mathrm{cm}}^{-1}{\;\mathrm{and}\;600\;\mathrm{cm}}^{-1}}\;}$$

where $A_{{966\;\mathrm{cm}}^{-1}}$ represents the integral area of butadiene (CH=CH) peaks near 966 cm^−1^, $A_{{1030\;\mathrm{cm}}^{-1}}$ denotes the integral area of sulfoxide (S=O) peaks near 1030 cm^−1^, $A_{{1600\;\mathrm{cm}}^{-1}}$ is the integral area of aromatic (C=C) peaks near 1600 cm^−1^, $A_{{1700\;\mathrm{cm}}^{-1}}$ is the integral area of aromatic (C=C) peaks near 1700 cm^−1^, and $\sum A_{{4000\;\mathrm{cm}}^{-1}{\;\mathrm{and}\;600\;\mathrm{cm}}^{-1}}\;$ is the integral area of all the characteristic peaks from 2000 cm^−1^ to 600 cm^−1^.

**Figure 3:**
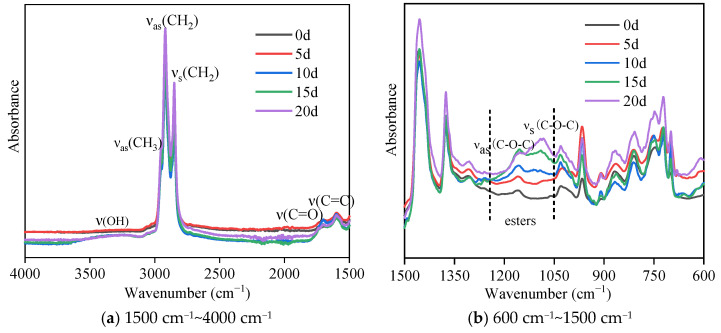
Infrared spectra of the SBR asphalt surface film at various aging durations.

Figure 3 presents the ATR-FTIR spectra of the SBR asphalt surface layer, highlighting significant changes in the carbonyl characteristic peak around 1700 cm^−1^ during the aging process. The SBR asphalt shows almost no characteristic carbonyl peak at 1700 cm^−1^ before aging; these results indicate that no carbonyl compounds were present in the original SBR asphalt. However, with the extension of the coupled aging time, the characteristic peak of the asphalt samples clearly appeared at 1700 cm^−1^ with aging times of 10 d, 15 d, and 20 d. The hydroxyl characteristic peak in the range of 3700~3110 cm^−1^ also significantly changed during the aging process. As aging progressed, the pattern of the hydroxyl characteristic peak changed from absent (0 d) to present (5 d), initially increasing (10 d and 15 d), and then decreasing (20 d). These results indicated that the aging process generated a large number of hydroxyl compounds, such as alcohols, ketones, acids, and esters. The characteristic ester doublet (1300–1000 cm^−1^) arose from coupled asymmetric stretching vibrations of the C-O-C group, confirming oxidative esterification during aging. The characteristic ester peak for the SBR asphalt in the range of 1300–1100 cm^−1^ during aging exhibited an initial increase (5 d and 10 d) followed by a decrease (15 d and 20 d). The carbonyl and ester peaks appeared later than the hydroxyl peak; this result reflected the dynamic changes in the compounds in the upper surface film layer during the coupled aging process. A large number of photo-oxidation products, such as alcohols, ketones, and acids, were generated in the earlier stages, whereas ester compounds are typically regarded as intermediate products of asphaltene. Ultimately, the hardened film layer on the surface of the asphalt should be formed by the condensation of photo-oxidation products, resulting in asphaltene or high polymers, as well as a small amount of inorganic compounds [36]. The results of the matrix asphalt are from our previous study [11]. Throughout the aging process, the change trends in the characteristic carbonyl, hydroxyl, and ester peaks were consistent for the SBR-modified asphalt and the matrix asphalt. However, a notable distinction between the SBR-modified asphalt and the matrix asphalt was the timing of the peak values for these characteristic peaks. For example, the ester peak in the broad range of approximately 1300–1100 cm^−1^ showed a noticeable increase for the matrix asphalt samples at 5 d and 10 d, but it gradually disappeared after 15 d and 20 d. In contrast, the SBR asphalt showed a significant increase at 15 d and 20 d. This study found that incorporating SBR into matrix asphalt postponed the aging process, as evidenced by the variation patterns and peak appearance times of carbonyl, hydroxyl, and ester peaks. This suggests that SBR enhances the asphalt’s resistance to ultraviolet aging, aligning with findings from a prior study [19].

**Figure 4:**
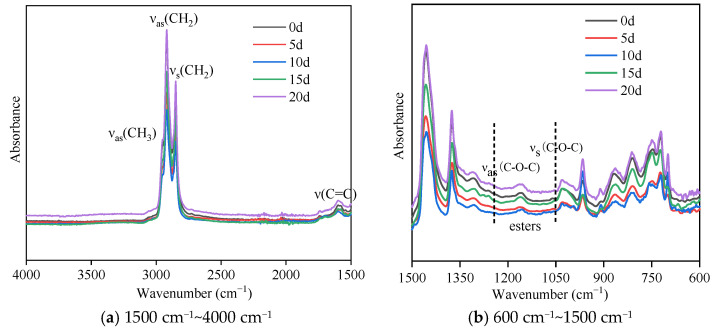
Infrared spectra of the lower layer of the SBR asphalt at various aging durations.

The infrared spectrum of the lower asphalt layer for the SBR-modified asphalt is shown in Figure 4, while the comparative matrix asphalt spectrum data were derived from our previous study [11]. Compared with the infrared spectra of the upper surface film layer of the asphalt, both types of lower asphalt did not exhibit the characteristic hydroxyl peak around 3700–3110 cm^−1^ and the characteristic carbonyl peak around 1700 cm^−1^; these results indicated that no relevant oxidation reactions occurred in the lower layers. The strong variations in the other characteristic peaks were not particularly evident, and their changes need further quantitative analysis for detailed interpretation.

**Figure 5:**
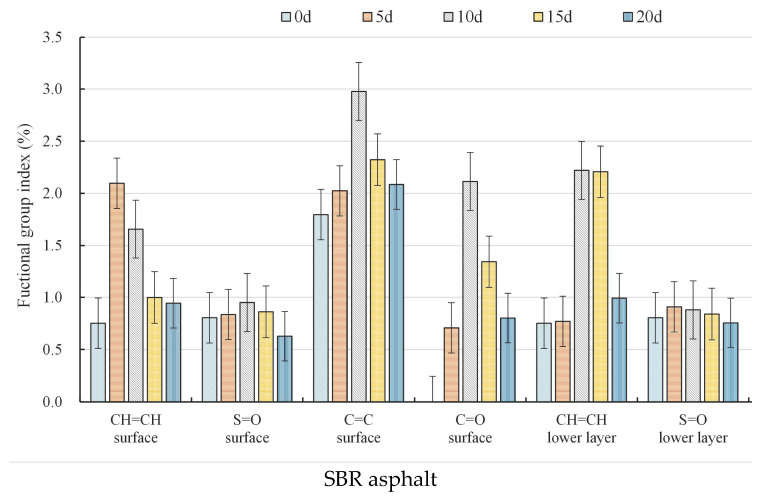
Typical functional group indexes of the SBR asphalt at various aging durations.

Figure 5 presents the variations in the functional group indices of both the surface film and the lower asphalt layer for the SBR-modified asphalt under coupled aging conditions, while the corresponding data for the matrix asphalt are available in our previous study [11]. The most notable distinction between the two kinds of asphalt was observed in the carbonyl (C=O) group. The variation trend in carbonyl (C=O) groups in the surface film of the SBR-modified asphalt is consistent with that of the matrix asphalt. However, after 20 d of aging, carbonyl compounds remained in the SBR-modified asphalt surface, suggesting a slower aging process compared to the matrix asphalt. The lower asphalt of both asphalt samples did not show any carbonyl groups; these results indicated that no oxidation reactions occurred in the lower asphalt of either the SBR or matrix asphalt.

The variation trend in the sulfoxides functional group (S=O) index for the surface film layer of the SBR asphalt initially increased at 5 d and 10 d and then decreased at 15 d and 20 d. Conversely, the lower layer asphalt showed an increase in the index after 5 d of aging, followed by a consistent decrease after 10 d, 15 d, and 20 d of aging. The variation trend in the sulfonyl functional groups (S=O) for the matrix asphalt was characterized by an increase at 5 d, a decrease at 10 d, another increase at 15 d, and a subsequent decrease at 20 d. In contrast, the lower asphalt increased at both 5 d and 10 d and then decreased at 15 d and 20 d. The observed variation trends indicated that the changes in the sulfone functional group indexes of the SBR asphalt occurred more slowly and exhibited smaller amplitude fluctuations with respect to the matrix asphalt. This was attributed to the cross-linking effect of the SBR modifier; this effect enhanced the stability of the asphalt structure and inhibited the generation and activity of free radicals. Consequently, the chain scission, decomposition, and oxidation processes on the asphalt surface layer were alleviated. The depolymerization, decomposition, and generation of new asphaltenes in the lower asphalt system were also suppressed to some extent under large temperature fluctuations, resulting in a reduction in the concentration of polar molecules. Moreover, the migration of polar molecules was constrained, and the migration rate of smaller asphaltene molecules decreased. Therefore, the sulfone functional group index values on the surface film did not exhibit sudden spikes.

The functional group index of the aromatic ring hydrocarbons (C=C) for the SBR asphalt surface film changed as follows: it increased during the first 5 d and 10 d, and then decreased at 15 d and 20 d. The overall variation pattern was consistent with that of the matrix asphalt. However, the matrix asphalt reached its maximum value after 5 d of aging, whereas the SBR asphalt reached its peak after 10d of aging. In the lower layer of the SBR asphalt, the aromatic ring hydrocarbon index continuously increased with increasing aging time, whereas the matrix asphalt reached its maximum index after 15 d of aging. These findings suggest that the incorporation of the SBR modifier significantly enhances the antiaging capacity of asphalt under the combined effects of intense UV radiation and large temperature differences. This enhancement primarily involved an inhibitory effect on ultraviolet-induced surface degradation and oxidation reactions, the formation of new asphaltenes in the lower asphalt system caused by large temperature differences, the depolymerization and decomposition of resins and asphaltenes, and the migration of polar compounds, thereby delaying the aging process of the asphalt.

Butadiene (CH=CH) is a distinctive functional group in SBR-modified asphalt. As coupled aging progressed, the functional group index of butadiene in both the surface and the lower layers of asphalt initially increased and then decreased. The values of the functional group indexes of butadiene for the surface layer began to decline after 10 d of aging, whereas that of the lower layer decreased after 15 d of aging. The decomposition of the SBR modifier particles on the surface layer under UV aging conditions led to an increase in the functional group index of butadiene, resulting in these observed results. Then, the aging promoted a more uniform distribution of SBR particles in the modified asphalt; this led to more SBR particles absorbing UV radiation, thereby significantly accelerating the increase rate of the butadiene index. Under continuous UV light, the double bonds in the butadiene molecules could absorb energy, causing the excitation of the molecules and the formation of free radicals. These free radicals initiated polymerization, causing butadiene molecules to combine into polymer compounds, thereby reducing the CH=CH functional group index. Therefore, the butadiene index of the upper surface film rapidly decreased under intense ultraviolet radiation. The lower layer of asphalt was decomposed mainly by the large temperature differences, and the decomposition process was relatively slow with respect to the surface layer. After decomposition was complete, the internal asphalt appeared to contain more free radicals, which led to other chemical reactions and a reduction in the functional group index of butadiene.

### 3.3. Molecular Size Evolution Analyses

**Figure 6:**
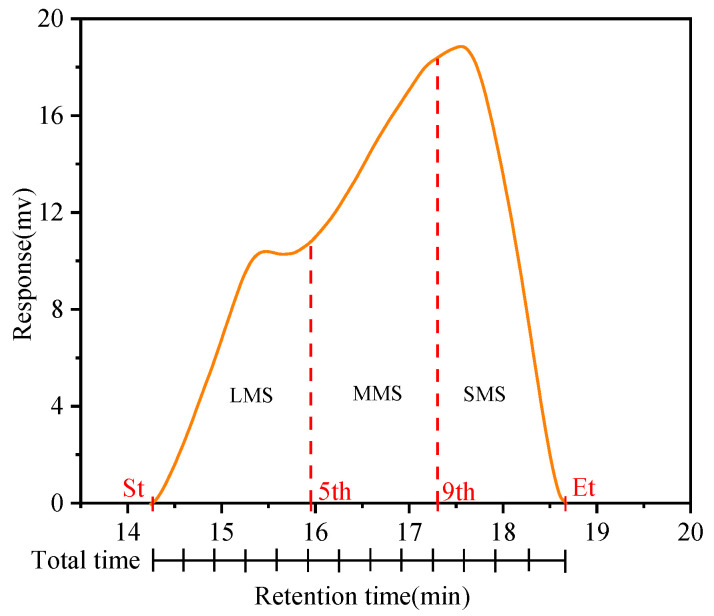
Methodology for analyzing molecular size.

Based on the GPC testing principle, each asphalt sample chromatogram was segmented into 13 slices with equal elution times. The initial 5 slices correspond to a large molecular size (LMS), the next 6–9 slices denote a medium molecular size (MMS), while the remaining 4 slices indicate a small molecular size (SMS) [37,38,39]. The GPC curves are shown in Figure 6 [29], and the three divisions are expressed as percentages. To further quantify the effects of coupled aging on the molecular system, this study adopted the increments of LMS, MMS, and SMS as evaluation indicators, with their specific calculations given in Equations (5)–(7)

(5) $$I_{LMS}=\frac{{\left(LMS\right)}_{\;aged}-{\left(LMS\right)}_{virgin}}{{\left(LMS\right)}_{virgin}}\times 100\%$$

(6) $$I_{MMS}=\frac{{\left(MMS\right)}_{\;aged}-{\left(MMS\right)}_{virgin}}{{\left(MMS\right)}_{virgin}}\times 100\%$$

(7) $$I_{SMS}=\frac{{\left(SMS\right)}_{\;aged}-{\left(SMS\right)}_{virgin}}{{\left(SMS\right)}_{virgin}}\times 100\%$$

**Figure 7:**
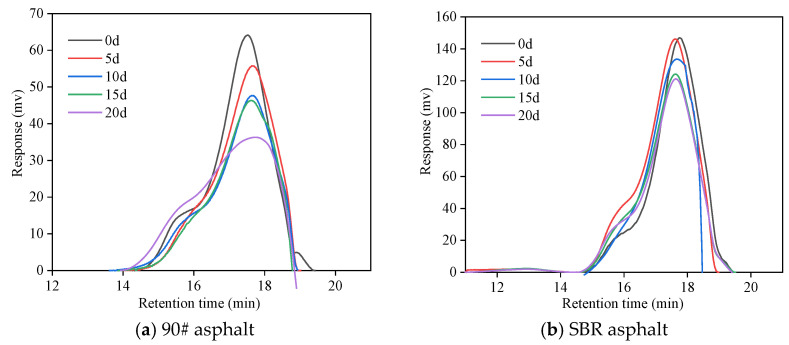
GPC curves of asphalt at various aging durations.

**Figure 8:**
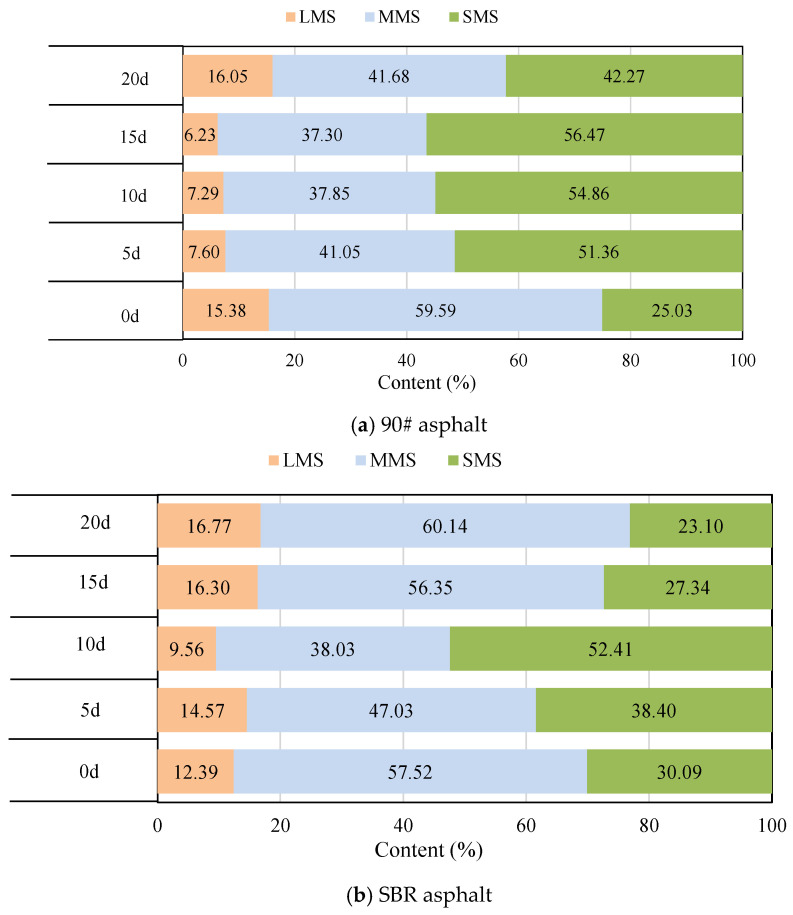
Molecular distribution of asphalt at various aging durations.

Figure 7 and Figure 8 illustrate notable differences in the molecular distributions and molecular weight distributions between the SBR asphalt and the matrix asphalt. After 5 d of aging, the GPC curve for the SBR-modified asphalt shifted toward higher molecular weights, with an increase in the LMS content, a decrease in the MMS content, and an increase in the SMS content. In contrast, the GPC curve of the matrix asphalt shifted toward a lower molecular weight, with a decrease in the LMS content. The variation patterns of the MMS and SMS were consistent with those of the SBR-modified asphalt, but with significantly greater increases. These results primarily occurred because the large and medium molecules of both asphalts underwent decomposition during the initial aging stage. However, the degradation of the SBR modifier compensated for the loss of high-molecular-weight components within the asphalt colloidal system, leading to a significant rise in the concentration of higher-molecular-weight LMS. Additionally, the presence of the SBR modifier effectively suppressed photodegradation and photo-oxidation processes on the upper surface layer of the asphalt; this significantly weakened the increases in the MMS and SMS contents. After 10 d of aging, the LMS of the SBR-modified asphalt significantly decreased, whereas the MMS decreased and SMS increased. Similarly, the LMS of the matrix asphalt also decreased, but its rate of decrease was noticeably reduced. These findings suggest that SBR significantly postponed the overall aging of the asphalt system.

After 15 d of aging, the SBR-modified asphalt exhibited an increase in LMS and MMS, while SMS decreased. The LMS and MMS of the matrix asphalt decreased, and the SMS increased. The main reason for the rapid increase in the LMS of the SBR asphalt was the reorganization and polymerization reactions of the small-molecule asphaltenes that migrated to the surface. Furthermore, the SBR asphalt showed characteristic ester peaks after aging for 15 d; these results indicated that the oxygen-containing compounds (such as hydroxyls, ketones, aldehydes, carboxylic acids, etc.) formed by photochemical oxidation further reacted to form esters or other compounds; this led to a sharp increase in the macromolecules and medium-sized molecules. Additionally, the matrix asphalt exhibited noticeable ester peaks after aging for 5 d; these results further demonstrated that the SBR modifier effectively delayed the aging process of the asphalt.

After 20 d of aging, the LMS of the SBR-modified asphalt continued to increase, but the rate of increase was significantly reduced. The MMS increased, whereas the SMS decreased. The LMS and MMS of the matrix asphalt increased, whereas the SMS decreased. The changes in the base asphalt were significantly greater than those in the SBR asphalt, indicating a higher aging degree in the base asphalt compared to the SBR asphalt. Throughout the aging process, the minimum LMS value for the SBR asphalt was 9.56%, whereas the minimum value for the base asphalt was 6.23%. The stability of the system is largely attributed to the cross-linking effect of the SBR modifier, which significantly reduces the degradation of asphalt macromolecules under the coupled effects. As a result, fewer compounds on the asphalt surface participated in photodecomposition and photo-oxidation reactions. Moreover, the degradation of the SBR modifier compensated for the lost components within the asphalt system, preserving its compositional equilibrium and effectively slowing down the aging process.

Table 2 presents the increments of parameters ($I_{LMS}$, $I_{MMS}$, $I_{SMS}$) at different aging times. The data demonstrate that the absolute increment values of all molecular fractions in the SBR-modified asphalt were consistently lower than those in the matrix asphalt throughout the aging process. Specifically, the maximum absolute increments for the SBR-modified asphalt reached 35.33% ($I_{LMS}$), 33.88% ($I_{MMS}$), and 74.16% ($I_{SMS}$), compared to 59.45% ($I_{LMS}$), 37.41% ($I_{MMS}$), and 125.59% ($I_{SMS}$) for the matrix asphalt. These results clearly indicated that the SBR-modified asphalt underwent relatively smaller molecular compositional changes during aging, confirming its superior stability in the colloidal system.

The molecular size and weight distribution during the coupled aging process reflected the dynamic balance of various chemical reactions within the asphalt colloidal system. Initially, aging primarily involved the breakdown of the SBR modifier. As the process progressed to the middle stage, the focus transitioned to further decomposition of SBR and the disintegration of the asphalt components with medium to large molecular weights. In the later stages of aging, the asphalt surface layer experienced photochemical oxidation, photopolymerization, and photo-induced cross-linking reactions, mainly involving the oxidation of medium- and low-molecular-weight components, as well as the evaporation of low molecular weight substances [40,41].

### 3.4. Microstructural Evolution

#### 3.4.1. Microtopography

**Figure 9:**
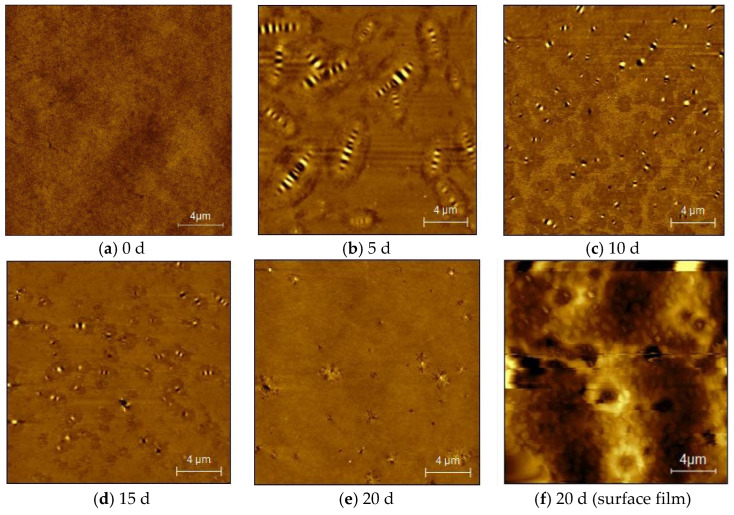
Micro-surface morphology of the SBR asphalt at various aging durations.

Figure 9 illustrates two-dimensional micro-surface morphology images of the lower-layer SBR asphalt at various aging durations and the surface film after 20 d of aging. A comparative analysis of the micromorphology between the SBR asphalt and the matrix asphalt [11] revealed insights into the aging mechanism. Notable differences were identified in the two-dimensional micromorphology between the upper surface film layer and the lower layer of the asphalt after 20 d of aging. The absence of a “bee structure” in the surface film suggested distinct chemical composition differences between the aging products in the film layer and the underlying asphalt layer. The surface exhibited numerous aggregates, as indicated by the black areas in the figure; these aggregates were presumed to originate from the asphaltenes and other oxidation products and exhibited strong polarity. The surface film was enriched with a significant amount of polar oxidation residues; these residues eventually separated from the nonpolar compounds and aggregated together, resulting in a distinct two-phase structure in the film layer. In contrast, the SBR asphalt had fewer aggregates with respect to the matrix asphalt. The photochemical cross-linking of the high-polymer-content surface film layer in the SBR asphalt was relatively lower in the later aging stages compared to the matrix asphalt, suggesting that the SBR asphalt’s aging process was delayed relative to the matrix asphalt’s aging process.

A comparative analysis highlighted notable micromorphological differences between the SBR-modified asphalt and the matrix asphalt, both before and after aging, providing insights into the effects of aging time. The “bee structure” of SBR exhibited significant alterations in both its quantity and area. The SBR-modified asphalt exhibited changes similar to the matrix asphalt only after aging, suggesting distinct aging pathways for the SBR-modified and matrix asphalt under coupled aging effects. The SBR-modified asphalt demonstrated a buffering effect during the initial aging stages, decelerating alterations in the “bee structure” characteristics compared to the matrix asphalt. The incorporation of the SBR modifier led to a mesh structure that significantly adsorbed asphaltene molecules, thereby delaying or inhibiting their decomposition. Additionally, in the later aging stages, the decomposition of the SBR modifier itself could replenish the content of large and small molecules within the asphalt, further delaying the aging process. Therefore, the SBR-modified asphalt exhibited superior antiaging performance with respect to the matrix asphalt. The “bee structure” effectively represents microscopic changes in both the matrix and SBR-modified asphalt before and after aging, indicating the asphalt’s antiaging capability [42].

#### 3.4.2. Micromechanics

**Figure 10:**
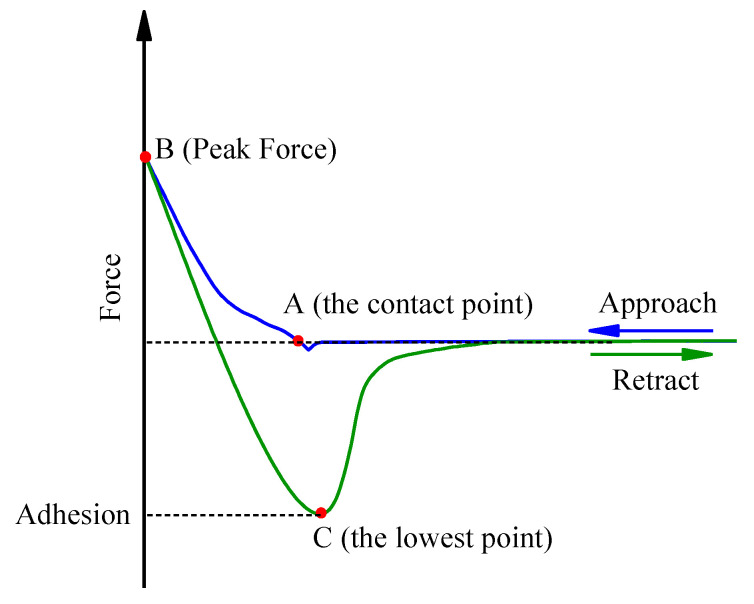
Typical AFM force–displacement (FD) curves.

This study utilized the microscopic Young’s modulus and adhesion force as mechanical indicators to examine the micromechanical properties of SBR asphalt throughout the aging process. A typical force‒displacement curve obtained from the AFM data of asphalt is illustrated in Figure 10 [11]. The adhesion force was calculated as the force difference between the contact and separation points [43,44]. The Young’s modulus was determined by employing the Derjaguin–Muller–Toporov (DMT) model from contact mechanics, which is suitable for fitting the probe retraction process, as outlined in Equations (8) and (9) [45].

(8) $$F_{tip}-F_{ad}=\frac{4}{3}E^{*}\sqrt{Rd^{3}}$$

(9) $$E^{*}={\left[\frac{1-V_{S}^{2}}{E_{S}}+\frac{1-V_{\mathrm{tip}}}{E_{tip}}\right]}^{-1}$$

where $F_{tip}$ represents the force exerted on the probe cantilever, nN; $F_{ad}$ denotes the adhesion force between the probe and the sample, nN; $E^{*}$ represents the sample’s effective modulus, MPa; R represents the probe tip’s radius, nm; d represents the indentation depth of the sample, nm; $V_{S}$ and $V_{tip}$ are the Poisson’s ratios of the sample and probe respectively; and $E_{S}$ and $E_{\mathrm{tip}}$ are the elastic Young’s modulus of the sample and the probe respectively, MPa.

**Figure 11:**
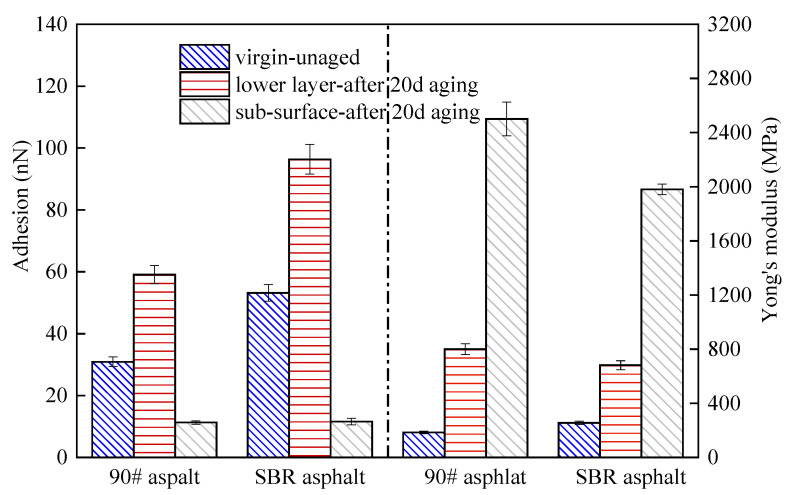
Adhesion force and Young’s modulus of asphalt before and after aging for 20 d.

Figure 11 presents the micromechanical analysis results of the unaged virgin asphalt and the asphalt aged for 20 days. The adhesion force and Young’s modulus of the matrix asphalt and the SBR asphalt exhibited consistent trends under the coupled effects. After 20 d of aging, the adhesive forces of the asphalt significantly decreased, particularly with the surface film’s adhesion force plummeting sharply to a low value, indicating poor adhesion performance; the Young’s modulus of the surface film layer was significantly higher than that of the unaged asphalt, indicating the film layer had become very hard under the coupled aging. The Young’s modulus of the underlying layer asphalt increased compared to the unaged asphalt, though the increase was not substantial. The observed patterns suggest that asphalt surface cracking is mainly due to property discrepancies between its upper and underlying layers.

**Figure 12:**
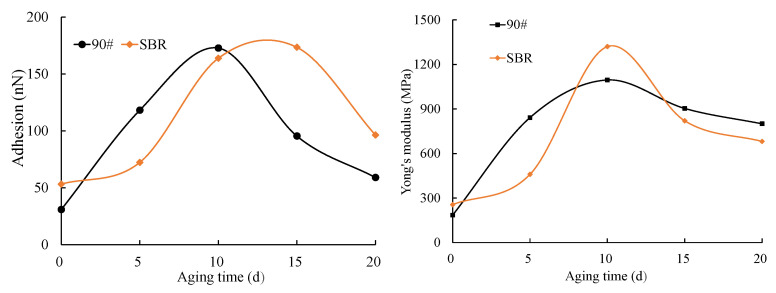
Adhesion force and Young’s modulus of the lower-layer asphalt at various aging durations.

To further substantiate this deduction, the changes in the lower layer of the asphalt with aging time were analyzed, and the results are presented in Figure 12. Quantitative analysis of the data in Figure 12 revealed that incorporating SBR into the matrix asphalt resulted in a 72.16% increase in the adhesion force and a 38.5% enhancement in Young’s modulus compared to the matrix asphalt. The enhancement was mainly due to the SBR modifier, which facilitated network structure formation in the asphalt colloidal system and improved the adhesion properties. The adhesion force and Young’s modulus of both the matrix and SBR asphalt initially increased and then decreased with aging time. However, the peak values for the SBR asphalt occurred later than those for the matrix asphalt, suggesting enhanced aging resistance. In the initial aging phases, both the adhesion force and Young’s modulus of the asphalt increased with aging time, aligning with findings from prior research [46,47,48]. The adhesion force and Young’s modulus of the asphalt’s underlying layer began to decrease with prolonged exposure to the combined effects, likely caused by the impact of asphaltenes on the adhesion properties of the asphalt [49]. Although the trends in the changes in the adhesion force and Young’s modulus for both the SBR and matrix asphalt were similar, some notable differences were observed. The matrix asphalt exhibited a significantly higher rate of increase in the adhesion force during early aging stages compared to the SBR asphalt, reaching its peak value sooner. This suggests that matrix asphalt is more sensitive to the coupled aging and ages more rapidly. The delayed aging of the SBR-modified asphalt is attributed to the SBR modifier’s absorption of lighter asphalt components by the SBR modifier upon its addition to the asphalt, which causes swelling that restricts molecular movement and forms a stable network structure. The stable network prevented macromolecule decomposition and small molecule migration, while the degradation of SBR increased small molecule numbers in the aging process, thus preserving the asphalt colloidal system’s stability.

However, the SBR-modified asphalt developed cracks earlier than the matrix asphalt; this was primarily related to the modulus gradient. As shown in Figure 12, after 20 d of aging, the modulus ratio of the surface film to the lower layer of the matrix asphalt was 3.13, while the modulus ratio of the surface film to the lower layer of for SBR asphalt was 2.91. More severe cracking was observed in the matrix asphalt than in the SBR-modified asphalt after 20 d of aging. Therefore, it can be concluded that a greater modulus ratio correlated with increased cracking severity; this conclusion will be further explored and validated in future studies. The earlier cracking of the SBR-modified asphalt is attributed to its lower Young’s modulus in the lower layer compared to the matrix asphalt after 15 d of aging, indicating a rapid decline from a higher modulus. This led to a swift increase in the modulus ratio for SBR and resulted in a localized concentration of temperature stress, which caused cracking. These results also indicated that a “critical value” for asphalt cracking existed. The smaller the difference in properties between the surface layer and the underlying asphalt during the aging process, the less likely it is to crack.

## 4. Conclusions

Intense ultraviolet radiation is often accompanied by significant temperature variations in high-altitude cold regions. Therefore, elucidating aging mechanisms under the coupled effects of strong UV radiation and large temperature differences is crucial for accurately analyzing the causes of asphalt pavement distress in these areas. This research utilized infrared spectroscopy, gel permeation chromatography (GPC), and atomic force microscopy (AFM) to examine the chemical composition, microstructure, and micromechanical characteristics of SBR asphalt as aging progressed. The objectives of this study were to reveal the aging behavior of SBR asphalt under the combined effects of intense UV radiation (irradiation intensity of 180 W/m^2^) and large temperature differences (−20 °C~50 °C). The following main conclusions were drawn from this research.

(1) Macromorphology analysis indicated that a high-temperature non-melting film layer formed on the asphalt surface under the coupled effects of intense UV radiation and large temperature differences. As aging progressed, the surface contraction increased, the film gloss was diminished, the color deepened, the surface became dry, the aging film layer thickened, and noticeable cracking occurred in the later stages.
(2) Changes in the chemical composition indicated that the overall pattern of chemical component evolution was consistent between the SBR asphalt and the matrix asphalt and primarily involved photo-oxidation, photodecomposition, polycondensation, and photoinduced crosslinking reactions. However, compared to those in the matrix asphalt, the peak values of the characteristic carbonyl, hydroxyl, and ester peaks in the SBR asphalt appeared significantly later. The carbonyl, sulfonyl, aromatic hydrocarbon, and butadiene indexes suggested a slower aging process for the SBR asphalt. Furthermore, the molecular weight distribution analysis indicated that the SBR-modified asphalt exhibited less variation in molecular composition compared to the matrix asphalt during aging. Specifically, the maximum absolute increments for the SBR-modified asphalt reached 35.33% ($I_{LMS}$), 33.88% ($I_{MMS}$), and 74.16% ($I_{SMS}$) compared to 59.45% ($I_{LMS}$), 37.41% ($I_{MMS}$), and 125.59% ($I_{SMS}$) for the matrix asphalt. These results indicated that SBR effectively enhanced the colloidal stability of the matrix asphalt, thereby suppressing the aging process.
(3) The AFM test data analysis revealed significant differences in the aging pathways between the SBR-modified asphalt and matrix asphalt. The SBR-modified asphalt showed consistent changes with the matrix asphalt only after a period of initial aging; these results indicated that the incorporation of the SBR modifier delayed the aging process. The network structure of the SBR-modified asphalt provided a buffering effect in the initial aging phase. The degradation of the SBR modifier subsequently mitigated changes in the “bee structure” characteristics. Additionally, a gradient aging phenomenon was clearly observed throughout the aging process.
(4) The analysis of the adhesion forces and Young’s modulus (DMT modulus) from the FD force curve indicated that gradient aging, influenced by the coupled effects, was the main factor causing crack formation from the surface layer downward. The risk of surface cracking can be quantitatively evaluated by the modulus ratio; a higher modulus ratio between the surface film and the underlying asphalt layer is associated with an elevated cracking risk and increased crack propagation rate. During the middle-to-late aging stages, the modulus of the lower layer in the SBR-modified asphalt decreased more significantly than in the matrix asphalt, leading to an increased stress concentration between the surface and underlying layers. As a consequence, the surface cracking of the SBR-modified asphalt occurs earlier than that of the matrix asphalt under the coupled effects of intense UV radiation and large temperature differences. This characteristic may induce premature initiation of top-down cracking in SBR-modified asphalt pavements during the early service period in high-altitude cold regions.

## 5. Future Research Work

This study reveals the aging evolution mechanism of SBR-modified asphalt under the combined effects of intense ultraviolet radiation and large temperature variations. However, this research is limited to indoor simulation tests, and the conclusions require validation through outdoor field tests. In future research, to more accurately replicate pavement aging in high-altitude cold regions, natural exposure aging tests of different types of asphalt (SBS asphalt and rubber asphalt) will be conducted in the Tibetan Plateau area to validate the reliability of indoor simulated aging tests and aging mechanisms. Additionally, the aging of various types of asphalt mixtures will also be studied, and the evolution patterns of the macroscopic and microscopic properties of asphalt along the depth direction will be investigated. A spatiotemporal evolution-based asphalt aging prediction model will be proposed.

## Figures and Tables

**Table 1:** Basic technical indexes of main materials.

Material Type	Technical Indexes
Shell90#	Penetration (25 °C, 5 s, 100 g): 88.4 (0.1 mm), ductility (5 mm/min 5 °C): 13.5 cm, softening point (ring and ball method): 47.7 °C, density (25 °C): 1.030 g/cm^3^, storage stability: 0.4 °C.
SBR	The polymer modifier SBR is a white powder, containing 23.0% styrene, with a tensile strength of 25 MPa, an elongation at break of 378%, and a volatile content of 0.6%.
SBR-modified asphalt	Penetration (25 °C, 5 s, 100 g): 76.0 (0.1 mm), ductility (5 mm/min 5 °C): >100 cm, softening point (ring and ball method): 52.3 °C, density (25 °C): 1.031 g/cm^3^, storage stability: 0.9 °C.

**Table 2:** Increments of each molecular fraction.

	Aging Time	5 d	10 d	15 d	20 d	Sample
Index						
$I_{LMS}$ (%)		−50.60	−52.56	−59.45	4.36	90#
		17.59	−22.82	35.33	31.58	SBR asphalt
$I_{MMS}$ (%)		−31.12	−36.49	−37.41	−30.05	90#
		−18.24	−33.88	4.55	−2.03	SBR asphalt
$I_{SMS}$ (%)		105.19	119.16	125.59	68.87	90#
		27.62	74.16	−23.25	−9.13	SBR asphalt

## Data Availability

The original contributions presented in this study are included in the article. Further inquiries can be directed to the corresponding author.
